# Does Moxa Smoke Have Significant Effect on the Acupuncturist's Respiratory System? A Population-Based Study

**DOI:** 10.1155/2019/4873235

**Published:** 2019-09-16

**Authors:** Chang Yu, Ning Zhang, Weikang Zhu, Yueyue Zhang, Jiao Yang, Yong Wang, Xiaoge Song, Ling Hu, Zijian Wu, Qi Liu, Yong Tang, Qiaofeng Wu, Shuguang Yu

**Affiliations:** ^1^Acupuncture and Moxibustion College, Chengdu University of Traditional Chinese Medicine, Chengdu, Sichuan 610075, China; ^2^CEMS, NCMIS, MDIS, Academy of Mathematics & Systems Science, Chinese Academy of Sciences, Beijing 100080, China; ^3^Acupuncture and Moxibustion College, Anhui University of Traditional Chinese Medicine, Hefei, Anhui 230000, China; ^4^Acupuncture and Moxibustion College, Shaanxi University of Traditional Chinese Medicine, Xi'an, Shaanxi 712046, China

## Abstract

**Objectives:**

To evaluate the safety of moxa smoke, especially to provide quantitative information and details for the occupational prevention of acupuncturists.

**Methods:**

We combined the questionnaire-based cross-sectional survey and lung function testing-based historical retrospective cohort research to investigate the safety of moxa smoke exposure (MSE) among acupuncturists. A mathematical regression model was established to quantitatively evaluate the relationship between moxa smoke exposure and the respiratory health of the acupuncturist. The smoke exposure time of the acupuncturist and the prevalence of abnormal respiratory symptoms or diseases were also evaluated.

**Results:**

(1) The cross-sectional research showed that the incidence of expectoration (18.7%) and rhinitis (22.7%) was the most common respiratory symptom and disease after MSE. No statistical difference was found between smoke exposure time of the acupuncturist and the prevalence of abnormal respiratory symptoms or diseases, except the prevalence of rhinitis and shortness of breath (*P* < 0.01). Regression model for the incidence of first three symptoms (expectoration, shortness of breath, and wheezing) from the cross-sectional survey indicated that the weight coefficients of factors associated with moxa smoke were lower than those of factors unrelated to moxa smoke, such as gender and personal history of respiratory diseases. (2) Historical retrospective cohort research showed that there was no significant difference in the % predicted PEF. No statistic difference was found between the exposed and nonexposed group in large airway function indexes (% predicted FEV_1_, % predicted FVC, and % predicted FEV_1_/FVC) and small airway function indexes (% predicted FEF_25_, % predicted FEF_50_, % predicted FEF_75_, and % predicted MMEF), either. Especially, the % predicted MVV among males (106.23 ± 2.92 vs. 95.56 ± 1.92, *P* < 0.01 and % predicted VC among females (100.70 ± 1.59 vs. 95.91 ± 1.61, *P* < 0.05) between the two groups had statistical significance, but did not cause pulmonary ventilation dysfunction.

**Conclusions:**

MSE has no significant effect on the respiratory health of acupuncturists.

## 1. Introduction

Moxibustion is one of the traditional Chinese medicine (TCM) therapies that use the heat generated by burning herbal preparations containing *Artemisia vulgaris* (mugwort) to stimulate acupuncture points [1]. In China and some other Asian countries, moxibustion has been used to treat various diseases such as painful disease [2], knee osteoarthritis [3, 4], gastrointestinal diseases [5], antiaging [6], immunomodulatory [7], primary dysmenorrhea [8], primary insomnia [9], chronic fatigue syndrome [10], and cancer-related fatigue [11]. Nowadays, moxibustion is being increasingly accepted as an alternative treatment for correct nonvertex presentation [12], irritable bowel syndrome [13], inflammation bowel disease [14], and neurological symptoms [15], and so on. Hence, moxibustion has attracted increasing interest in more and more countries.

With people paying more attention to environmental pollution and their own health, the security challenge caused by moxa smoke has become the focus of attention, just as incense burning produces large amounts of particulate matter (PM), nitrogen dioxide, sulfur dioxide, formaldehyde, benzene, polycyclic aromatic hydrocarbons (PAH), and so on [16]. During the past decade, a considerable number of clinical and experimental studies have indicated that moxa smoke contains a range of chemical components, including inhalable particles (PM 10 and PM 2.5), formaldehyde, naphthalene, benzene, methylbenzene, total volatile organic compounds, CO, CO_2_, NO, SO_2_, NH_3_, and O_3_ [17, 18]. However, some studies have demonstrated that the concentrations of these substances are minimal and can be controlled within a safe concentration range in clinical [17, 19]. By keeping ventilation well, the contents of CO, NO_2_, PM 10, and PM 2.5 in the air can be controlled within safe ranges [20]. And under normal operating conditions, neither volatile nor carbon monoxide would do harm to health and safety [21]. Based on these studies, the potential effect of moxa smoke on health is controversial.

In previous studies, we found that moxa smoke causes adverse stimulus reactions to the body and affects the compliance of patients with moxibustion [22, 23]. Besides, a number of researchers have also shown solicitude for the safety of people exposed to moxa smoke. For example, Wang et al.'s research demonstrated that the body's responses of participants after exposed to moxa smoke were most notable in areas with exposed mucous membranes, such as the eyes, nose, and throat [24]. Zhao et al. focused on the patients' heart rate and heart rate variability after MSE [25]. However, most of them were focused on patients, and little work has been undertaken with regard to whether moxa smoke is harmful to acupuncture practitioners who were frequently exposed to moxa smoke. Hence, the present study aimed at achieving the following: (1) a cross-sectional survey was carried out among acupuncture practitioners, by using the American Thoracic Society Division of Lung Disease questionnaire (ATS-DLD-78-A), to observe the incidence of abnormal respiratory symptoms and respiratory diseases after MSE. Additionally, a corresponding mathematical model was established to quantitatively analyze the relationship between MSE and the respiratory health of acupuncturist using the data collected from the cross-sectional survey. (2) A historical retrospective cohort study was carried out on the lung function health status in the moxa smoke-exposed and nonexposed group using portable pulmonary function instrument (ST-75).

## 2. Methods

### 2.1. Study Design and Population

#### 2.1.1. The Cross-Sectional Survey

The cross-sectional survey was conducted between May 2016 and April 2017 to assess the respiratory health status of acupuncturists who practice acupuncture treatment regularly. Participants were recruited from 79 TCM hospitals, 30 prefectures (states and cities), spanning 4 provinces (including Sichuan province, Shaanxi province, Anhui province, and Hunan province) in China via face-to-face interview. Participants were enrolled if they had obtained the qualification of a Chinese medicine practitioner or assistant Chinese medicine practitioner; registered or engaged in acupuncture and moxibustion for at least one year or regularly conduct acupuncture and moxibustion diagnosis and treatment activities in TCM hospitals; between 23 and 70 years of age; and signed informed consent. The main exclusion criteria were long-term use of smokeless moxibustion or smokeless moxibustion appliances and acupuncture and moxibustion practice for less than one year.

The prevalence of respiratory health was assessed through the American Thoracic Society Division of Lung Disease adult questionnaire (ATS-DLD-78-A) which had been translated into Chinese. The questionnaire was translated by a professional translator, and another translator was asked to reassess its accuracy. They jointly determined the final Chinese version. The questionnaire has also been used in other respiratory health surveys in China [26, 27]. A structured questionnaire was used to obtain detailed information on sociodemographic characteristics, occupational factors, and confounding factors. Occupational factors contain some moxa exposure information, such as working years of moxibustion, average days of moxibustion activities per week, average number of moxibustion patients per day, average duration of receiving moxibustion therapy per patient, whether there was ventilation equipment in moxibustion clinic, subjective evaluation of moxibustion concentration in moxibustion environment, area of moxibustion clinic, and number of patients receiving treatment in the same moxibustion room at the same time. Confounding factors contain personal history of respiratory diseases, family history of respiratory diseases, smoking, second-hand smoke exposure, and so on.

The items of respiratory symptoms and diseases consisted of cough, chronic cough, phlegm, chronic phlegm, gasp for breath, wheezing, breathlessness, dyspnea on exertion, chest colds, chest illness, bronchitis, pneumonia, rhinitis, and chronic bronchitis, which were determined on the basis of positive answers to the following items:

  Cough: “Do you usually have a cough?” or “Do you often cough in the morning or when you get up?” Or “Do you often cough during the day or at night?”
  Chronic cough: “Do you usually cough four to six times a day, four days a week or more?” or “Do you usually cough like this for three months or more in a year?”
   Expectoration: “Do you often expectorate?” or “Do you often expectorate when you get up in the morning? Or it is the first thing in the morning?” or “Do you often expectorate during the day or at night?”
  Chronic expectoration: “Do you often expectorate twice a day, four days a week or more?” or “Do you have this kind of expectoration for three months or more in a year?”
  Gasp for breath: “Does your chest ever wheeze or whistle when you have a cold, or when you don't catch a cold, or do you make a sound of whistling most of the day or night?”
  Wheezing: “Have you ever had an attack of wheezing that has made you feel short of breath?”
  Shortness of breath: “Are you troubled by shortness of breath when hurrying on the level or walking up a slight hill?”
  Bronchitis, pneumonia, rhinitis, chronic bronchitis, asthma, and emphysema: “Have you ever had bronchitis/pneumonia/rhinitis/chronic bronchitis/asthma/emphysema and been confirmed by a doctor?”

A subgroup analysis was also conducted between smoke exposure time of the acupuncturist and abnormal respiratory symptoms and disease prevalence. Additionally, the corresponding mathematical model was established to quantitatively analyze the relationship between MSE and the respiratory health of acupuncturists using the data of the first three symptoms collected from the cross-sectional survey.

#### 2.1.2. Historical Retrospective Cohort Research

The cohort study was conducted between June 2016 and March 2018 to review the lung function between the group exposed to moxa smoke and nonexposed group, by using portable spirometer (Spiroanalyzer, ST-75, Fukuda Sangyo, Japan). Participants were recruited from 16 TCM hospitals in Chongqing city and Sichuan province of China. Cluster sampling method was used in this study. 142 acupuncturists were recruited as the observation group. The inclusion and exclusion criteria were consistent with the previous test. The control group was 142 nonacupuncturists (hospital administrators or other medical staff) who were not exposed to moxa smoke. According to the ratio of 1:1, matched by gender, average age difference was ＜3 year old, average height difference was <3 cm, and average weight difference was <5 kg. After filling out the questionnaires of sociodemographic data, the lung function indexes of all subjects were tested by portable spirometer, including mean percentage predicted vital capacity (VC), maximal ventilatory volume (MVV), peak expiratory flow (PEF), forced expiratory volume in the first second (FEV_1_), forced vital capacity (FVC), forced expiratory flow between 25% and 75% of the FVC (FEF_25–75%_), and maximum midexpiratory flow (MMEF).

Lung function measurements were performed with the assistance of a trained, skilled physician under supervision according to the American Thoracic Society guidelines [28]. For the purpose of quality assurance, spirograms were reviewed by a pulmonologist before inclusion in the study. The mean percentage predicted value was based on individual age, weight, height, and gender as calculated and adjusted by the spirometer device. Subjects were asked not to smoke for at least 1 h before testing. Additionally, the procedure was explained to participants and they were asked to rest in a sitting position until they felt comfortable. Results of three acceptable maneuvers were performed, and the best of the three readings was used for further analysis.

### 2.2. Quality Control

Three postgraduate students majored in acupuncture (two of them were investigators and one was a supervisor) who received the formal training of investigating were assigned to conduct the interviews. To ensure the quality of investigation, the supervisor conducted a spot check on the completeness of questionnaires. Ten percent of the respondents were randomly selected and asked to fill in the same questionnaire through a telephone survey to verify whether their responses after 15 days were consistent with those after the first face-to-face interview. It is unacceptable that the deviations were 10% or above.

### 2.3. Statistical Analysis

The collected data were put into a database by two investigators and analyzed using SPSS statistics version 21.0. The prevalence of respiratory symptoms and diseases was calculated by dividing the number of individuals who responded positive by the number of questionnaires completed. Demographic characteristics are presented as numbers, mean, and standard deviation (SD). Normality was checked firstly for all data of lung function, and then the comparisons of lung function between the exposed and nonexposed group were made by *t*-test. Data were expressed as mean ± SD. The level of significance was set at *P* < 0.05. The association between moxa smoke exposure and the respiratory health of the acupuncturist was analyzed by mathematical quantitative regression models.

## 3. Results

### 3.1. Incidence of the Acupuncturist's Abnormal Respiratory Symptoms and Respiratory Diseases after MSE

#### 3.1.1. Demographic Characteristics

A total of 825 acupuncture practitioners consented to participate in the study. After logical screening and data cleaning, 803 copies of valid data (322 males and 481 females) were obtained.  Table 1 shows the details of the demographic characteristics.

#### 3.1.2. Incidence of Abnormal Respiratory Symptoms and Respiratory Diseases after MSE

**Figure 1:**
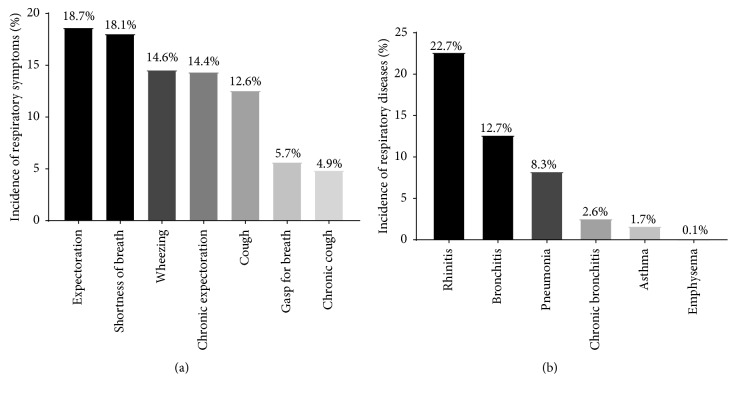
Incidence of abnormal respiratory symptoms and respiratory diseases after MSE. (a) Incidence of abnormal respiratory symptoms after MSE. The highest was expectoration (18.7%), followed by shortness of breath (18.1%), wheezing (14.6%), chronic expectoration (14.4%), cough (12.6%), gasp for breath (5.7%), and chronic cough (4.9%). (b) Incidence of respiratory diseases after MSE. The highest was rhinitis (22.7%), followed by bronchitis (12.7%), pneumonia (8.3%), chronic bronchitis (2.6%), asthma (1.7%), and emphysema (0.1%).

Among the 803 acupuncturists been surveyed, the incidence of expectoration (18.7%) was the highest in abnormal respiratory symptoms, followed by shortness of breath (18.1%), wheezing (14.6%), chronic expectoration (14.4%), cough (12.6%), gasp for breath (5.7%), and chronic cough (4.9%). Among the respiratory diseases, the incidence of rhinitis (22.7%) was the highest, followed by bronchitis (12.7%), pneumonia (8.3%), chronic bronchitis (2.6%), asthma (1.7%), and emphysema (0.1%) (Figure 1).

### 3.2. Subgroup Analysis

For the 803 acupuncturists, we split moxa smoke exposure time into total working years and moxa smoke average exposure each day and analyzed the different moxa smoke exposure time and abnormal respiratory symptoms and diseases prevalence. It can be seen from Table 2 that the prevalence of rhinitis in different working years groups and shortness of breath in groups of average exposure each day had a statistically significant difference (*P* < 0.01).

### 3.3. Quantitative Model of the Effect of Moxa Smoke on the Respiratory Health of Acupuncturists

In this study, a regression model was established for the incidence of the top three symptoms from the cross-sectional survey. All predictive variables were entered and passed through logistic stepwise regression (sig < 0.05 into the equation, sig > 0.10 removed from the equation) to obtain the results. From the regression results, the regression equation of the probability of suffering from expectoration/shortness of breath/wheezing and the predictive variable group *X* could be obtained; that is, *P*(*Y*=1 | *X*) was obtained.

#### 3.3.1. Quantitative Model Prediction of Expectoration

(1) $$\begin{array}{c} P\left(\text{expectoration}\right)=-0.962+1.892\;*\text{ Rhinitis}+3.561\;*\text{ Asthma}+0.614\;*\text{ Female}+15.506\;*\text{ Family history of maternal lung cancer.} \end{array}$$

It could be seen from this regression equation that the variables associated with moxa smoke were not contained in the model, which indicated that moxa smoke-related factors were not significant effective factors on the occurrence of expectoration. The bigger the coefficients in front of the variables are, the greater the predictive power of the occurrence of symptoms will be.

#### 3.3.2. Quantitative Model Prediction of Shortness of Breath

(2) PShortness of breath=−3.625+2.026 ∗ Rhinitis+1.631 ∗ Second−hand smoke exposure+2.52 ∗ Female+0.103 ∗ Minimal concentration+0.363 ∗ Smaller concentration+0.635 ∗ Medium concentration+2.265 ∗ Mother's chronic bronchitis+1.093 ∗ MSE.

The regression model showed that although MSE and the concentration of MSE were included in the regression equation, compared with other factors unrelated to moxa smoke, such as rhinitis, second-hand smoke exposure, and so on, the weight coefficients of the effect on shortness of breath of factors related to moxa smoke were much smaller. That is, it is limited to impact the shortness of breath for factors related to moxa smoke.

#### 3.3.3. Quantitative Model Prediction of Wheezing

(3) $$\begin{array}{c} P\left(\text{Wheezing}\right)=-2.137+2.580\;*\text{ Pneumonia}+1.781\;*\text{ Rhinitis}+5.631\;*\text{ chronic bronchitis}+1.684\;*\text{ Smoking}+0.438\;*\text{ Medium concentration.} \end{array}$$

It can be seen from this regression that the personal history of pneumonia, rhinitis, chronic bronchitis, smoking, and MSE concentration, and so on have a certain impact on the prediction of wheezing. Among them, the coefficient before the four variables of pneumonia, rhinitis, chronic bronchitis, and smoking was bigger; however, the coefficient before the variable of moxa smoke concentration was smaller. Obviously, among the factors affecting wheezing, factors unrelated to moxa smoke account for a larger proportion.

### 3.4. Lung Function Comparison of Acupuncturists between Moxa Smoke-Exposed Group and Nonexposed Group

#### 3.4.1. Demographic Characteristics

284 acupuncture practitioners consented to participate in the study.  Table 3 shows the demographic characteristics of the survey population. Demographics, including gender (*χ*^2^ = 0.127, *P* > 0.05), age distribution (*χ*^2^ = 5.368, *P* > 0.05), smoking status (*χ*^2^ = 0.230, *P* > 0.05), and BMI (*χ*^2^ = 4.638, *P* > 0.05) as indicated, did not differ between the two groups (*P* > 0.05).

#### 3.4.2. Lung Function Comparison between the Moxa Smoke-Exposed and Nonexposed Group

**Figure 2:**
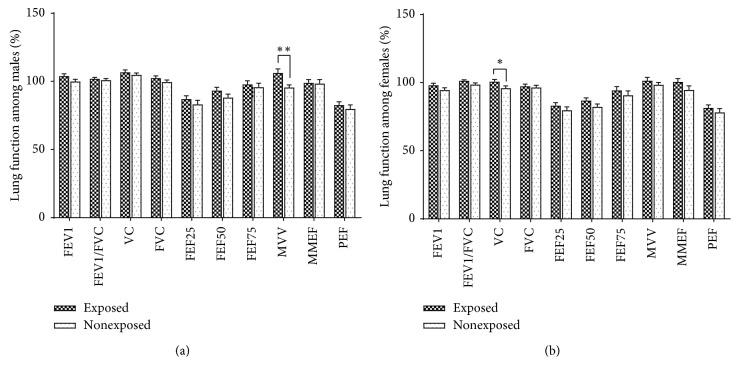
Lung function comparison between the moxa smoke-exposed and nonexposed group. (a) Male lung function indexes comparison between the exposed and nonexposed group. Compared with the nonexposed group, the % predicted MVV index had statistical significance (106.23 ± 2.92 vs. 95.56 ± 1.92, *P* < 0.01). (b) Female lung function indexes comparison between the exposed and nonexposed group. Compared with the nonexposed group, the % predicted VC index had statistical significance (100.70 ± 1.59 vs. 95.91 ± 1.61, *P* < 0.05).

The lung function indexes comparison of the moxa smoke-exposed and nonexposed group is displayed in Table 4. When comparing the two groups, there were no significant differences in the % predicted PEF, large airway function indexes (% predicted FEV_1_, % predicted FVC, and % predicted FEV_1_/FVC), and small airway function indexes (% predicted FEF_25_, % predicted FEF_50_, % predicted FEF_75_, and % predicted MMEF). However, the % predicted MVV among males (106.23 ± 2.92 vs. 95.56 ± 1.92, *P* < 0.01) and % predicted VC among females (100.70 ± 1.59 vs. 95.91 ± 1.61, *P* < 0.05) between the exposed and nonexposed group had statistical significance, but it did not cause pulmonary ventilation dysfunction (Table 4 and Figure 2).

## 4. Discussion

This epidemiological study combined cross-sectional survey and historical retrospective cohort research, focusing on the clinical safety of moxa smoke among acupuncturists. A mathematical model for predicting the respiratory health of acupuncturists was specially established. This strategy can more comprehensively demonstrate the correlation between MSE and respiratory symptoms and diseases of acupuncturists and quantitatively analyze the effects of moxa smoke on the respiratory health of acupuncturists.

The safety evaluation research of moxa smoke has been deeply studied from the aspects of component analysis, toxicological mechanism, environmental monitoring, environmental toxicity, and so on; however, the conclusions are not unanimous. In order to further search for safety evidence of moxibustion, experts proposed to expand the scope of the investigation, carry out large-scale epidemiological research, and provide direct evidence for clinical safety evaluation of moxa smoke [29]. Additionally, in view of the increasing concern about environmental pollution, little work has been undertaken with regard to whether moxa smoke is harmful to acupuncturists who frequently exposed to moxa smoke. A moxibustion frontline workers investigation is especially necessary. Xu et al. found that cough and dry eyes were the main adverse stimulus reactions after MSE [30]. It was partly in line with the research by our previous investigation [22]. Therefore, the epidemiological investigation on the safety evaluation of moxa smoke should especially focus on the effect of moxa smoke on the respiratory system.

In this epidemiological study, we found that the incidence of expectoration and rhinitis was the most common respiratory symptom and disease after MSE. After subgroup analysis, we observed that except the prevalence of rhinitis and shortness of breath, no statistical difference was found between different MSE time of the acupuncturist and the prevalence of wheezing, chronic expectoration, cough, gasp for breath, chronic cough, bronchitis, pneumonia, chronic bronchitis, asthma, and emphysema. However, the quantitative mathematical model showed that the respiratory health of acupuncturists was more closely related to their own histories of respiratory diseases, family history of respiratory diseases, gender, smoking status, and so on. In some other studies, Li found that long-term inhalation of moxa smoke can lead to increased fatigue among healthcare workers [31]. Han et al. found that high concentration of moxa smoke condensation showed toxicity to induce chromosome damage, which disappears at low concentration [32]. Additionally, at a certain concentration of 9–12 mg/m^3^, there was no significant effect on people's respiratory rate, blood pressure, heart rhythm, degree of blood oxygen saturation, and other physiological indicators [33]. Considering the complexity of moxibustion process, whether long-term exposure to moxibustion can lead to diseases is controversial. Data from Xu's research demonstrated that the position, duration, distance between moxa and skin, proficiency of doctors, patient conditions, stimulations from smoke, and even the environment of treatment could affect the safety evaluation of moxibustion. The exact causes of most of these adverse events cannot be determined [30]. This is consistent with the opinions of most acupuncture clinical experts. In the course of this study, we learned that most acupuncturists who have worked for more than 20 years and frequently exposed to moxa smoke did not feel any obvious adverse respiratory reaction caused by long-term exposure to moxa smoke.

The historical retrospective cohort research showed that there was no significant difference in the % predicted PEF; in addition, large airway function indexes (% predicted FEV_1_, % predicted FVC, and % predicted FEV_1_/FVC) and small airway function indexes (% predicted FEF_25_, % predicted FEF_50_, % predicted FEF_75_, and % predicted MMEF) between the exposed and nonexposed group did not differ, either. Recently, animal research conducted by He et al. also demonstrated that the FEV1/FVC%, inspiratory resistance, and expiratory resistance among each group after 24 weeks of MSE had no difference. It was also mentioned that MSE at low concentrations did not affect the rat's lung function and moxa smoke of low concentrations (27.45 mg/m^3^) is much higher than that in a regular moxibustion clinic (3.54 mg/m^3^) [34]. Furthermore, animal research carried out in another study indicated that long-term MSE with medium and high concentration may cause inflammatory changes in the lung and bronchi, but it was not obvious in low concentration. No significant changes were found in the FEV%, FVC%, FEV 0.3/FVC, MMEF%, and PEF% of lung function in the three concentration groups, indicating that long-term continuous moxibustion with high concentration of moxa smoke could cause some pathological changes in lung and bronchial tissues, but it has no definite effect on lung function [35]. This is basically consistent with what we have observed in clinical practice. Strikingly, for different genders, there were some differences between the exposed and nonexposed group. Statistical significances were found in the % predicted MVV index among males and % predicted VC index among females between the exposed and nonexposed group, while it did not cause pulmonary ventilation dysfunction. The reason why the association of lung function differed in men and women is uncertain, but it may relate to their different sensitivities. Additionally, the greater number of men with MSE would provide a more precise measurement of calculated lung function than in women and would be more likely to be able to detect the small differences noted.

In this study, the mathematical model of quantitative analysis was used to evaluate the relationship between MSE and the respiratory health of the acupuncturist. It has not been mentioned in other similar studies. ATS-DLD-78-A was formulated by the American Thoracic Society (ATS) in 1978. It combines the previous investigation contents and experience of the British Medical Research Council (BMRC) and the National Heart and Lung Institute (NHLI), which is widely used in the epidemiological investigation of respiratory diseases in the general population or occupational population [26, 27, 36]. In addition, the cross-sectional survey belongs to the descriptive research method and the cohort study belongs to the analytical research method, while both of them belong to the observational research method in epidemiological research methods. Commonly, cross-sectional studies should be conducted to determine whether exposure factors are associated with certain health outcomes, and then cohort studies should be used to prove the causal relationship between them. It is through cohort studies that the BMRC has confirmed the link between doctors' smoking and lung cancer [37].

Interestingly, there is also reason to suspect that women may respond differently than men when exposed to the same MSE risk factors. In addition, their own history of respiratory diseases had a great influence on the safety evaluation of moxa smoke. Therefore, it is necessary to strengthen the protection of acupuncturists with a history of respiratory diseases in clinic.

There are several limitations in the present study. In view of the weak foundation of epidemiological research on moxa smoke, cross-sectional surveys and historical retrospective cohort studies have been carried out in this study. However, in order to provide more reliable evidence and form a relatively complete epidemiological evidence chain, it is necessary to carry out prospective cohort studies in the next step. At the same time, we did not test the representative objective indicators of the respiratory system in the cross-sectional survey, such as lung function. Additionally, in the process of research, we should also strengthen the consideration of exposure duration, exposure concentration, and so on.

## 5. Conclusion

Moxa smoke exposure has no significant effect on the respiratory health of acupuncturists. The regression model showed that the weight coefficients of moxa smoke factors were limited. The predictive effect of moxa smoke factors on the respiratory health of acupuncturists was less significant. Our findings therefore support that the occurrence of respiratory symptoms and diseases of acupuncturists was more closely related to their own history of respiratory diseases, family history of respiratory diseases, gender, smoking status, and so on.

## Figures and Tables

**Table 1:** Demographic characteristics.

	Frequency (*n*), mean ± SD	Constituent ratio (%)
Gender		
Male	322	40.1
Female	481	59.9
Age, mean		
Male	33.05 ± 0.443	
Female	30.74 ± 0.346	
Age distribution		
20∼	393	49.1
30∼	273	34.0
40∼	101	12.6
50∼	33	4.1
Average body mass index (BMI)		
Underweight	72	9.0
Normal	589	73.3
Overweight	119	14.8
Obesity	23	2.9
Degree of education		
Below undergraduate	44	5.5
Undergraduate and above	759	94.5
Ethnic		
Han nationality	782	97.5
Others	20	2.5
Marital status		
Unmarried	289	36
Married	514	64
Smoking status		
Smoking	92	11.5
Used to smoke	31	3.9
Never smoke	680	84.6
Second-hand smoke exposure		
No	227	28.3
Yes	576	71.7

SD, standard deviation.

**Table 2:** Subgroup analysis: correlation between different MSE time of acupuncturists and abnormal respiratory symptoms and diseases prevalence.

		Cough (%)	Chronic cough (%)	Expectoration (%)	Chronic expectoration (%)	Wheezing (%)	Gasp for breath (%)	Shortness of breath (%)	Bronchitis (%)	Pneumonia (%)	Rhinitis (%)	Chronic bronchitis (%)	Emphysema (%)	Asthma (%)
Working years (years)	1∼5	13.4	4.9	19.0	14.5	14.5	4.5	17.2	11.4	8.9	19.4	2.0	NA	1.3
	6∼10	8.6	3.4	19.4	13.1	14.3	7.4	21.1	15.4	6.3	24.6	1.7	NA	1.1
	11∼20	11.1	4.4	18.9	16.7	14.4	8.9	12.2	15.6	7.8	26.7	5.6	NA	2.2
	21∼	20.7	8.6	22.4	19.0	13.8	8.6	24.1	12.1	5.3	39.7	3.4	1.7	3.9
	*P* value	0.091	0.456	0.940	0.692	0.997	0.206	0.181	0.472	0.600	0.004^*∗*^	0.237	0.158	0.104

Average exposure each day (d·person·min)	1∼30	11.3	3.9	17.8	11.7	14.8	3.9	15.7	10.0	7.0	22.2	1.3	NA	1.3
	31∼120	15.6	5.0	19.4	17.5	13.1	7.5	8.1	10.0	6.3	21.9	3.1	NA	2.5
	121∼150	15.3	6.3	20.7	16.2	17.1	9.9	23.4	10.8	9.0	27.0	0.9	NA	2.7
	150∼	10.9	5.0	18.2	14.2	14.2	4.6	23.2	16.9	10.3	21.9	4.0	0.3	1.3
	*P* value	0.363	0.810	0.917	0.413	0.830	0.086	0.001^*∗*^	0.053	0.389	0.704	0.159	0.646	0.637

*Notes*. Average moxa smoke exposure each day = (average days of moxibustion activities per week (d) ^*∗*^average number of patients per day (person) ^*∗*^average duration of receiving moxibustion therapy per patient (min)) ÷ 7. ^*∗*^*P* < 0.05 was recognized as significant difference between groups.

**Table 3:** Demographic characteristics comparison between the moxa smoke-exposed and nonexposed group.

	Exposed group		Nonexposed group	
	Male (*n*, %)	Female (*n*, %)	Male (*n*, %)	Female (*n*, %)
Gender	73 (51.4)	69 (48.6)	73 (51.4)	69 (48.6)
Age distribution				
25–34	37 (26.1)	36 (25.4)	37 (26.1)	36 (25.4)
35–44	22 (15.5)	20 (14.1)	22 (15.5)	20 (14.1)
45–54	13 (9.2)	11 (7.7)	13 (9.2)	11 (7.7)
≥55	2 (1.4)	1 (0.7)	2 (1.4)	1 (0.7)
Smoking status				
Smoking	22 (15.5)	0	22 (15.5)	0
Never smoke	51 (35.9)	69 (48.6)	51 (35.9)	69 (48.6)
BMI				
Underweight	2 (1.4)	8 (5.6)	5 (3.5)	14 (9.9)
Normal	37 (26.1)	53 (37.3)	38 (26.8)	43 (30.1)
Overweight	26 (18.3)	7 (4.9)	25 (17.6)	12 (8.5)
Obesity	8 (5.6)	1 (0.7)	5 (3.5)	0

**Table 4:** Lung function comparison between the moxa smoke-exposed and nonexposed group.

Variable	Exposed group (mean ± SD)	Nonexposed group (mean ± SD)
*Male*		
*n* = 146		
% predicted FEV_1_	103.98 ± 1.59	99.93 ± 1.67
% predicted FEV_1_/FVC	101.99 ± 0.94	100.92 ± 1.25
% predicted VC	106.64 ± 1.75	104.84 ± 1.35
% predicted FVC	102.39 ± 1.69	99.50 ± 1.59
% predicted FEF_25_	87.11 ± 2.38	83.14 ± 3.03
% predicted FEF_50_	93.37 ± 2.36	88.13 ± 2.54
% predicted FEF_75_	97.78 ± 2.62	95.75 ± 2.99
% predicted MVV	106.23 ± 2.92^*∗∗*^	95.56 ± 1.92
% predicted MMEF	98.89 ± 2.37	98.43 ± 2.91
% predicted PEF	82.70 ± 2.34	79.80 ± 3.03

*Female*		
*n* = 138		
% predicted FEV_1_	98.05 ± 1.51	94.57 ± 1.57
% predicted FEV_1_/FVC	101.25 ± 0.83	98.59 ± 1.16
% predicted VC	100.70 ± 1.59^*∗*^	95.91 ± 1.61
% predicted FVC	97.29 ± 1.69	96.43 ± 1.60
% predicted FEF_25_	83.11 ± 2.18	79.64 ± 2.49
% predicted FEF_50_	86.78 ± 1.98	82.15 ± 2.18
% predicted FEF_75_	94.35 ± 2.70	90.69 ± 3.27
% predicted MVV	101.39 ± 2.46	98.39 ± 1.85
% predicted MMEF	100.61 ± 2.35	94.67 ± 2.84
% predicted PEF	81.48 ± 2.25	78.22 ± 2.73

^*∗*^ *P* < 0.05 and ^*∗∗*^*P* < 0.01 compared with the nonexposed group.

## Data Availability

The data used to support the findings of this study are available from the corresponding author upon request.
